# Classical Statistics and Statistical Learning in Imaging Neuroscience

**DOI:** 10.3389/fnins.2017.00543

**Published:** 2017-10-06

**Authors:** Danilo Bzdok

**Affiliations:** ^1^Department of Psychiatry, Psychotherapy and Psychosomatics, Medical Faculty, RWTH Aachen University, Aachen, Germany; ^2^Translational Brain Medicine, Jülich-Aachen Research Alliance (JARA), Aachen, Germany; ^3^Parietal Team, Institut National de Recherche en Informatique et en Automatique (INRIA), Gif-sur-Yvette, France

**Keywords:** neuroimaging, data science, epistemology, statistical inference, machine learning, *p*-value, Rosetta Stone

## Abstract

Brain-imaging research has predominantly generated insight by means of classical statistics, including regression-type analyses and null-hypothesis testing using *t*-test and ANOVA. Throughout recent years, statistical learning methods enjoy increasing popularity especially for applications in rich and complex data, including cross-validated out-of-sample prediction using pattern classification and sparsity-inducing regression. This concept paper discusses the implications of inferential justifications and algorithmic methodologies in common data analysis scenarios in neuroimaging. It is retraced how classical statistics and statistical learning originated from different historical contexts, build on different theoretical foundations, make different assumptions, and evaluate different outcome metrics to permit differently nuanced conclusions. The present considerations should help reduce current confusion between model-driven classical hypothesis testing and data-driven learning algorithms for investigating the brain with imaging techniques.

“The trick to being a scientist is to be open to using a wide variety of tools.”

*Breiman (2001)*

## Introduction

Among the greatest challenges humans face are cultural misunderstandings between individuals, groups, and institutions (Hall, 1976). The topic of the present paper is the culture clash between knowledge generation based on null-hypothesis testing and out-of-sample pattern generalization (Friedman, 1998; Breiman, 2001; Shmueli, 2010; Donoho, 2015). These statistical paradigms are now increasingly combined in brain-imaging studies (Kriegeskorte et al., 2009; Varoquaux and Thirion, 2014). Ensuing inter-cultural misunderstandings are unfortunate because the invention and application of new research methods has always been a driving force in the neurosciences (Greenwald, 2012; Yuste, 2015). Here the goal is to disentangle the contexts underlying *classical statistical inference* and *out-of-sample generalization* by providing a direct comparison of their historical trajectories, modeling philosophies, conceptual frameworks, and performance metrics.

**Figure 1:**
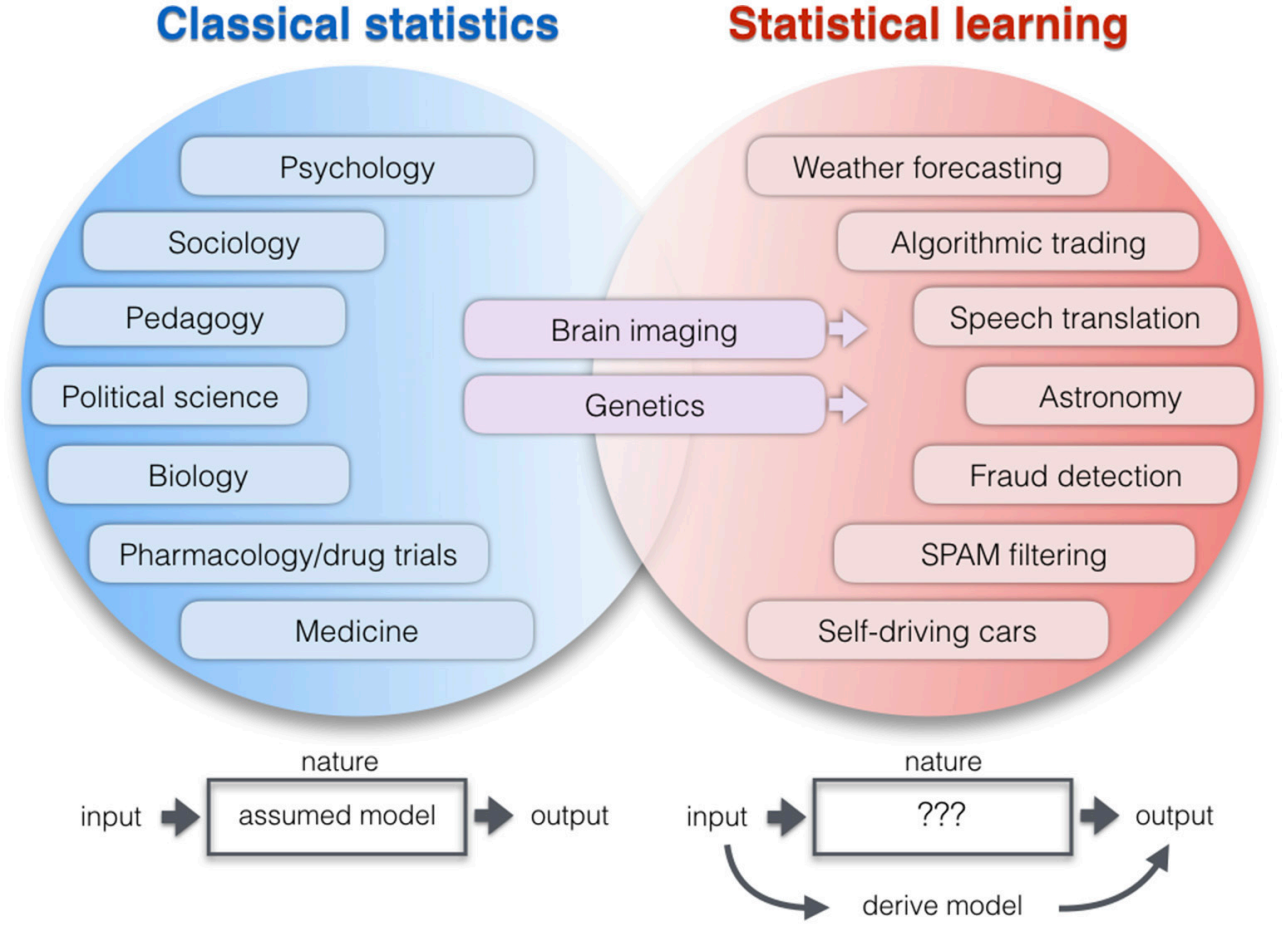
Application areas of two statistical paradigms. Lists examples of research domains which apply relatively more classical statistics (blue) or learning algorithms (red). The co-occurrence of increased computational resources, growing data repositories, and improving pattern-learning techniques have initiated a shift toward less hypothesis-driven and more algorithmic methodologies. As a broad intuition, researchers in the empirical sciences on the left tend to use statistics to evaluate a pre-assumed model on the data. Researchers in the application domains on the right tend to derive a model directly from the data: A new function with potentially many parameters is created that can predict the output from the input alone without explicit programming model. One of the key differences becomes apparent when thinking of the neurobiological phenomenon under study as a black box (Breiman, 2001). ClSt typically aims at modeling the black box by making a set of formal assumptions about its content, such as the nature of the signal distribution. Gaussian distributional assumptions have been very useful in many instances to enhance mathematical convenience and, hence, computational tractability. Instead, StLe takes a brute-force approach to model the output of the black box (e.g., tell healthy and schizophrenic people apart) from its input (e.g., volumetric brain measurements) while making a possible minimum of assumptions (Abu-Mostafa et al., 2012). In ClSt the stochastic processes that generated the data is therefore treated as partly known, whereas in StLe the phenomenon is treated as complex, largely unknown, and partly unknowable.

During recent years, neuroscience has transitioned from qualitative reports of few patients with neurological brain lesions to quantitative lesion-symptom mapping on the voxel level in hundreds of patients (Gläscher et al., 2012). We have gone from manually staining and microscopically inspecting single brain slices to 3D models of neuroanatomy at micrometer scale (Amunts et al., 2013). We have also gone from experimental studies conducted by a single laboratory to automatized knowledge aggregation across thousands of previously isolated neuroimaging findings (Yarkoni et al., 2011; Fox et al., 2014). Rather than laboriously collecting in-house data published in a single paper, investigators are now routinely reanalyzing multi-modal data repositories (Derrfuss and Mar, 2009; Markram, 2012; Van Essen et al., 2012; Kandel et al., 2013; Poldrack and Gorgolewski, 2014). The detail of neuroimaging datasets is hence growing in terms of information resolution, sample size, and complexity of meta-information (Van Horn and Toga, 2014; Eickhoff et al., 2016; Bzdok and Yeo, 2017). As a consequence of the data demand of many pattern-recognition algorithms, the scope of neuroimaging analyses has expanded beyond the predominance of regression-type analyses combined with null-hypothesis testing (Figure 1). Applications of statistical learning methods (i) are more data-driven due to particularly flexible models, (ii) have scaling properties compatible with high-dimensional data with myriads of input variables, and (iii) follow a heuristic agenda by prioritizing useful approximations to patterns in data (Jordan and Mitchell, 2015; LeCun et al., 2015; Blei and Smyth, 2017). *Statistical learning* (Hastie et al., 2001) henceforth comprises the umbrella of “machine learning,” “data mining,” “pattern recognition,” “knowledge discovery,” “high-dimensional statistics,” and bears close relation to “data science.”

From a technical perspective, one should make a note of caution that holds across application domains such as neuroscience: While the research question often precedes the choice of statistical model, perhaps no single criterion exists that alone allows for a clear-cut distinction between classical statistics and statistical learning in all cases. For decades, the two statistical cultures have evolved in partly independent sociological niches (Breiman, 2001). There is currently a scarcity of scientific papers and books that would provide an explicit account on how concepts and tools from classical statistics and statistical learning are exactly related to each other. Efron and Hastie are perhaps among the first to discuss the issue in their book “Computer-Age Statistical Inference” (2016). The authors cautiously conclude that statistical learning inventions, such as support vector machines, random-forest algorithms, and “deep” neural networks, can not be easily situated in the classical theory of twentieth century statistics. They go on to say that “pessimistically or optimistically, one can consider this as a bipolar disorder of the field or as a healthy duality that is bound to improve both branches” (Efron and Hastie, 2016, p. 447). In the current absence of a commonly agreed-upon theoretical account from the technical literature, the present concept paper examines applications of classical statistics vs. statistical learning in the concrete context of neuroimaging analysis questions.

More generally, ensuring that a statistical effect discovered in one set of data extrapolates to new observations in the brain can take different forms (Efron, 2012). As one possible definition, “the goal of statistical inference is to say what we have learned about the population *X* from the observed data x” (Efron and Tibshirani, 1994). In a similar spirit, a committee report to the National Academies of the USA stated (Committee on the Analysis of Massive Data et al., 2013, p. 8): “Inference is the problem of turning data into knowledge, where knowledge often is expressed in terms of variables […] that are not present in the data *per se*, but are present in models that one uses to interpret the data.” According to these definitions, *statistical inference can be understood as encompassing not only the classical null-hypothesis testing framework but also Bayesian model inversion to compute posterior distributions as well as more recently emerged pattern-learning algorithms relying on out-of-sample generalization* (cf. Gigerenzer and Murray, 1987; Cohen, 1990; Efron, 2012; Ghahramani, 2015). The important consequence for the present considerations is that classical statistics and statistical learning can give rise to different categories of inferential thinking (Chamberlin, 1890; Platt, 1964; Efron and Tibshirani, 1994)—an investigator may ask an identical neuroscientific question in different mathematical contexts.

For a long time, knowledge generation in psychology, neuroscience, and medicine has been dominated by classical statistics with *estimation* of linear-regression-like models and subsequent *statistical significance testing* whether an effect exists in the sample. In contrast, computation-intensive pattern learning methods have always had a strong focus on *prediction* in frequently extensive data with more modest concern for interpretability and the “right” underlying question (Hastie et al., 2001; Ghahramani, 2015). In many statistical learning applications, it is standard practice to quantify the ability of a predictive pattern to extrapolate to other samples, possibly in individual subjects. In a two-step procedure, a learning algorithm is fitted on a typically bigger amount of available data (*training data*) and the ensuing fitted model is empirically evaluated on a commonly smaller amount of independent data (*test data*). This stands in contrast to classical statistical inference where the investigator seeks to reject the null hypothesis by considering the entirety of a data sample (Wasserstein and Lazar, 2016), typically all available subjects. In this case, the desired relevance of a statistical relationship in the underlying population is ensured by formal mathematical proofs and is not commonly ascertained by explicit evaluations on new data (Breiman, 2001; Wasserstein and Lazar, 2016). As such, generating insight according to classical statistics and statistical learning serves rather distinct modeling purposes. Classical statistics and statistical learning do therefore not judge data on the same aspects of evidence (Breiman, 2001; Shmueli, 2010; Arbabshirani et al., 2017; Bzdok and Yeo, 2017). The two statistical cultures perform different types of principled assessment for successful extrapolation of a statistical relationship beyond the particular observations at hand.

Taking an epistemological perspective helps appreciating that scientific research is rarely an entirely objective process but deeply depends on the beliefs and expectations of the investigator. A new “scientific fact” about the brain is probably not established in vacuo (Fleck et al., 1935; terms in quotes taken from source). Rather, a research “object” is recognized and accepted by the “subject” according to socially conditioned “thought styles” that are cultivated among members of “thought collectives.” A witnessed and measured neurobiological phenomenon tends to only become “true” if not at odds with the constructed “thought history” and “closed opinion system” shared by that subject. The present paper will revisit and reintegrate two such thought milieus in the context of imaging neuroscience: classical statistics (ClSt) and statistical learning (StLe).

## Different histories: the origins of classical hypothesis testing and pattern-learning algorithms

**Figure 2:**
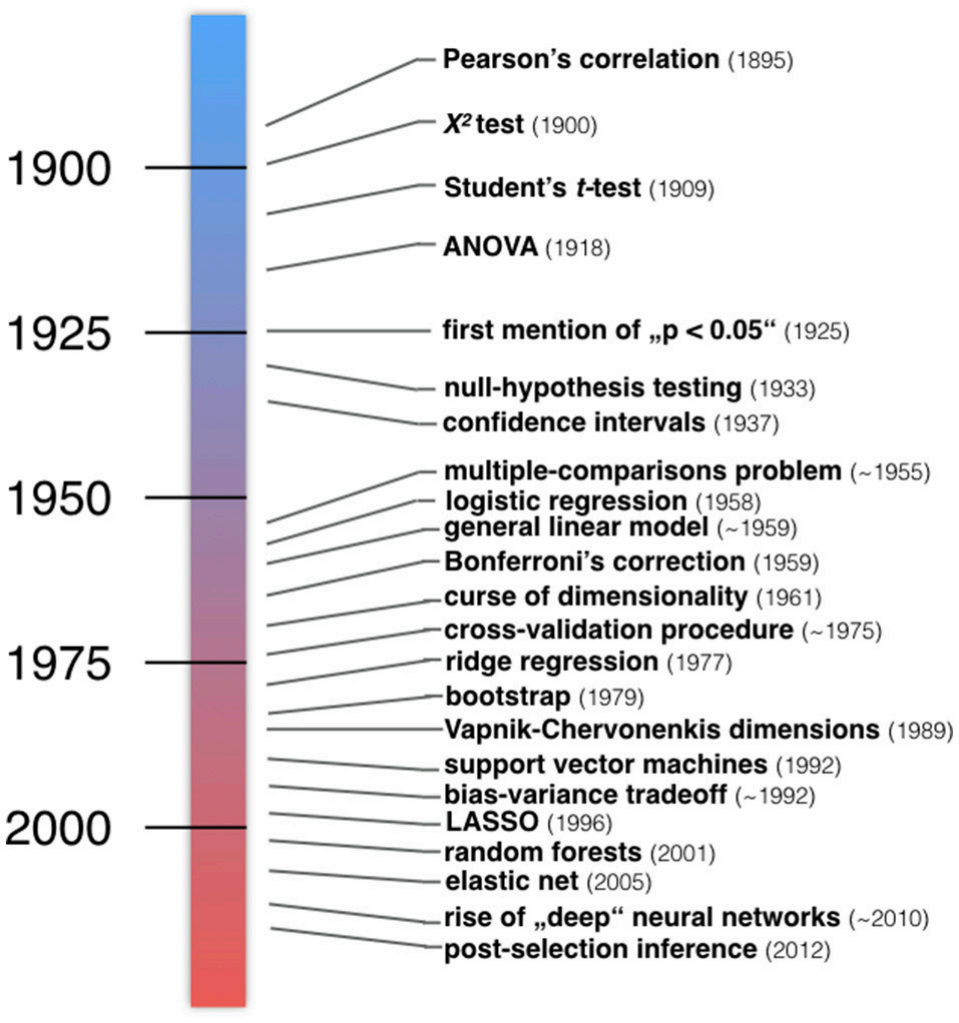
Developments in the history of classical statistics and statistical learning. Examples of important inventions in statistical methodology. Roughly, a number of statistical methods taught in today's textbooks in psychology and medicine have emerged in the first half of the twentieth century (blue). Instead, many algorithmic techniques and procedures have emerged in the second half of the twentieth century (red). “The postwar era witnessed a massive expansion of statistical methodology, responding to the data-driven demands of modern scientific technology.” (Efron and Hastie, 2016).

One of many possible ways to group statistical methods is by framing them along the lines of ClSt and StLe. The incongruent historical developments of the two statistical communities are even evident from their basic terminology. Inputs to statistical models are usually called *independent variables, explanatory variables*, or *predictors* in the ClSt community, but are typically called *features* collected in a *feature space* in the StLe community. The model outputs are typically called *dependent variables, explained variable*, or *responses* in ClSt, while these are often called *target variables* in StLe. It follows a summary of characteristic events in the development of what can today be considered as ClSt and StLe (Figure 2).

Around 1900 the notions of *standard deviation, goodness of fit*, and the *p* < 0.05 threshold emerged (Cowles and Davis, 1982). This was also the period when William S. Gosset published the *t*-test under the incognito name “Student” to quantify production quality in Guinness breweries. Motivated by concrete problems such as the interaction between potato varieties and fertilizers, Ronald A. Fisher invented the *analysis of variance* (ANOVA), *null-hypothesis testing*, promoted *p-values*, and devised principles of proper experimental conduct (Fisher and Mackenzie, 1923; Fisher, 1925, 1935). Another framework by Jerzy Neyman and Egon S. Pearson proposed the *alternative hypothesis*, which allowed for the statistical notions of *power, false positives* and *false negatives*, but left out the concept of *p*-values (Neyman and Pearson, 1933). This was a time before electrical calculators emerged after World War II (Efron and Tibshirani, 1991; Gigerenzer, 1993). Student's *t*-test and Fisher's inference framework were institutionalized by American psychology textbooks widely read in the 40s and 50s, while Neyman and Pearson's framework only became increasingly known in the 50s and 60s. Today's applied statistics textbooks have inherited a mixture of the Fisher and Neyman-Pearson approaches to statistical inference.

It is a topic of current debate^1^^,^^2^^,^^3^ whether ClSt is a discipline that is separate from StLe (e.g., Chambers, 1993; Breiman, 2001; Friedman, 2001; Bishop and Lasserre, 2007; Shalev-Shwartz and Ben-David, 2014; Efron and Hastie, 2016) or if “statistics” denotes a broader methodological class that includes both ClSt and StLe tools as its members (e.g., Tukey, 1962; Cleveland, 2001; Jordan and Mitchell, 2015; Blei and Smyth, 2017). StLe methods may be more often adopted by computer scientists, physicists, engineers, and others who typically have less formal statistical background and may be more frequently working in industry rather than academia. In fact, John W. Tukey foresaw many of the developments that led up to what one might today call statistical learning (Tukey, 1962). He early proposed a “peaceful collision of computing and statistics”. A modern reformulation of the same idea states (Efron and Hastie, 2016): “If the inference/algorithm race is a tortoise-and-hare affair, then modern electronic computation has bred a bionic hare.” Indeed, kernel methods, decision trees, nearest-neighbor algorithms, graphical models, and various other statistical tools actually emerged in the ClSt community, but largely continued to develop in the StLe community (Friedman, 2001).

As often cited beginnings of statistical learning approaches, the *perceptron* was an early brain-inspired computing algorithm (Rosenblatt, 1958), and Arthur Samuel created a checker board program that succeeded in beating its own creator (Samuel, 1959). Such studies toward *artificial intelligence* (AI) led to enthusiastic optimism and subsequent periodes of disappointment during the so-called “AI winters” in the late 70s and around the 90s (Russell and Norvig, 2002; Kurzweil, 2005; Cox and Dean, 2014), while the increasingly available computers in the 80s encouraged a new wave of statistical algorithms (Efron and Tibshirani, 1991). Later, the use of StLe methods increased steadily in many quantitative scientific domains as they underwent an increase in data richness from classical “long data” (samples *n* > variables *p*) to increasingly encountered “wide data” (*n* < < *p*) (Tibshirani, 1996; Hastie et al., 2015). The emerging field of StLe has received conceptual consolidation by the seminal book “The Elements of Statistical Learning” (Hastie et al., 2001). The coincidence of changing data properties, increasing computational power, and cheaper memory resources encouraged a still ongoing resurge in StLe research and applications approximately since 2000 (Manyika et al., 2011; UK House of Common S.a.T, 2016). For instance, over the last 15 years, *sparsity* assumptions gained increasing relevance for statistical and computational tractability as well as for domain interpretability when using *supervised* and *unsupervised* learning algorithms (i.e., with and without target variables) in the high-dimensional “*n* < < *p*” setting (Bühlmann and Van De Geer, 2011; Hastie et al., 2015). More recently, improvements in training very “deep” (i.e., many non-linear hidden layers) neural-networks architectures (Hinton and Salakhutdinov, 2006) have much improved automatized feature selection (Bengio et al., 2013) and have exceeded human-level performance in several application domains (LeCun et al., 2015).

In sum, “the biggest difference between pre- and post-war statistical practice is the degree of automation” (Efron and Tibshirani, 1994) up to a point where “almost all topics in twenty-first-century statistics are now computer-dependent” (Efron and Hastie, 2016). ClSt has seen many important inventions in the first half of the twentieth century, which have often developed at statistical departments of academic institutions and remain in nearly unchanged form in current textbooks of psychology and other empirical sciences. The emergence of StLe as a coherent field has mostly taken place in the second half of the twentieth century as a number of disjoint developments in industry and often non-statistical departments in academia (e.g., AT&T Bell Laboratories), which lead for instance to artificial neural networks, support vector machines, and boosting algorithms (Efron and Hastie, 2016). Today, systematic education in StLe is still rare at the large majority of universities, in contrast to the many consistently offered ClSt courses (Cleveland, 2001; Vanderplas, 2013; Burnham and Anderson, 2014; Donoho, 2015).

In neuroscience, the advent of brain-imaging techniques, including positron emission tomography (PET) and functional magnetic resonance imaging (fMRI), allowed for the *in-vivo* characterization of the neural correlates underlying sensory, cognitive, or affective tasks. Brain scanning enabled *quantitative* brain measurements with *many variables per observation* (analogous to the advent of high-dimensional microarrays in genetics; Efron, 2012). Since the inception of PET and fMRI, deriving topographical localization of neural activity changes was dominated by analysis approaches from ClSt, especially the general linear model (Scheffé, 1959; Poline and Brett, 2012; GLM). The classical approach to neuroimaging analysis is probably best exemplified by the statistical parametric mapping (SPM) software package that implements the GLM to provide a mass-univariate characterization of regionally specific effects.

As distributed information over voxels is less well captured by many ClSt approaches, including common GLM applications, StLe models were proposed early on for neuroimaging investigations. For instance, principal component analysis was used to distinguish globally distributed neural activity changes (Moeller et al., 1987) as well as to study Alzheimer's disease (Grady et al., 1990). Canonical correlation analysis was used to quantify complex relationships between task-free neural activity and schizophrenia symptoms (Friston et al., 1992). However, these first approaches to “multivariate” brain-behavior associations did not ignite a major research trend (cf. Worsley et al., 1997; Friston et al., 2008). As a seminal contribution, Haxby and colleagues devised an innovative across-voxel correlation analysis to provide evidence against the widely assumed face-specificity of neural responses in the ventral temporal cortex (2001). This ClSt realization of one-nearest neighbor classification based on correlation distance foreshadowed several important developments, including (i) joint analysis of sets of brain locations to capture “distributed and overlapping representations”, (ii) repeated analysis in different splits of the data sample to compare against chance performance, and (iii) analysis across multiple stimulus categories to assess the specificity of neural responses. The finding of distributed face representation was confirmed in independent, similar data (Cox and Savoy, 2003) and based on neural network algorithms (Hanson et al., 2004).

The application of StLe methods in neuroimaging increased further after rebranding as “mind-reading,” “brain decoding,” and “MVPA” (Haynes and Rees, 2005; Kamitani and Tong, 2005). Note that “MVPA” initally referred to “multi*voxel* pattern analysis” (Kamitani and Tong, 2005; Norman et al., 2006) and later changed to “multi*variate* pattern analysis” (Haynes and Rees, 2005; Hanke et al., 2009; Haxby, 2012). Up to that point, the term *prediction* had less often been used by imaging neuroscientists in the sense of out-of-sample generalization of a learning algorithm and more often in the incompatible sense of (in-sample) linear correlation such as using Pearson's or Spearman's method (Shmueli, 2010; Gabrieli et al., 2015). While there was scarce discussion of the position of “decoding” models in formal statistical terms, growing interest was manifested in first review publications and tutorial papers on applying StLe methods to neuroimaging data (Haynes and Rees, 2006; Mur et al., 2009; Pereira et al., 2009). The interpretational gains of this new access to the neural representation of behavior and its disturbances in disease was flanked by the availability of necessary computing power and memory resources. Although challenging to realize, “deep” neural network algorithms have recently been introduced to neuroimaging research (Plis et al., 2014; de Brebisson and Montana, 2015; Güçlü and van Gerven, 2015). These computation-intensive models might help in approximating and deciphering the nature of neural processing in brain circuits (Cox and Dean, 2014; Yamins and DiCarlo, 2016). As the dimensionality and complexity of neuroimaging datasets are constantly increasing, neuroscientific investigations will be always more likely to benefit from StLe methods given their natural scaling to large-scale data analysis (Efron, 2012; Efron and Hastie, 2016; Blei and Smyth, 2017).

**Figure 3:**
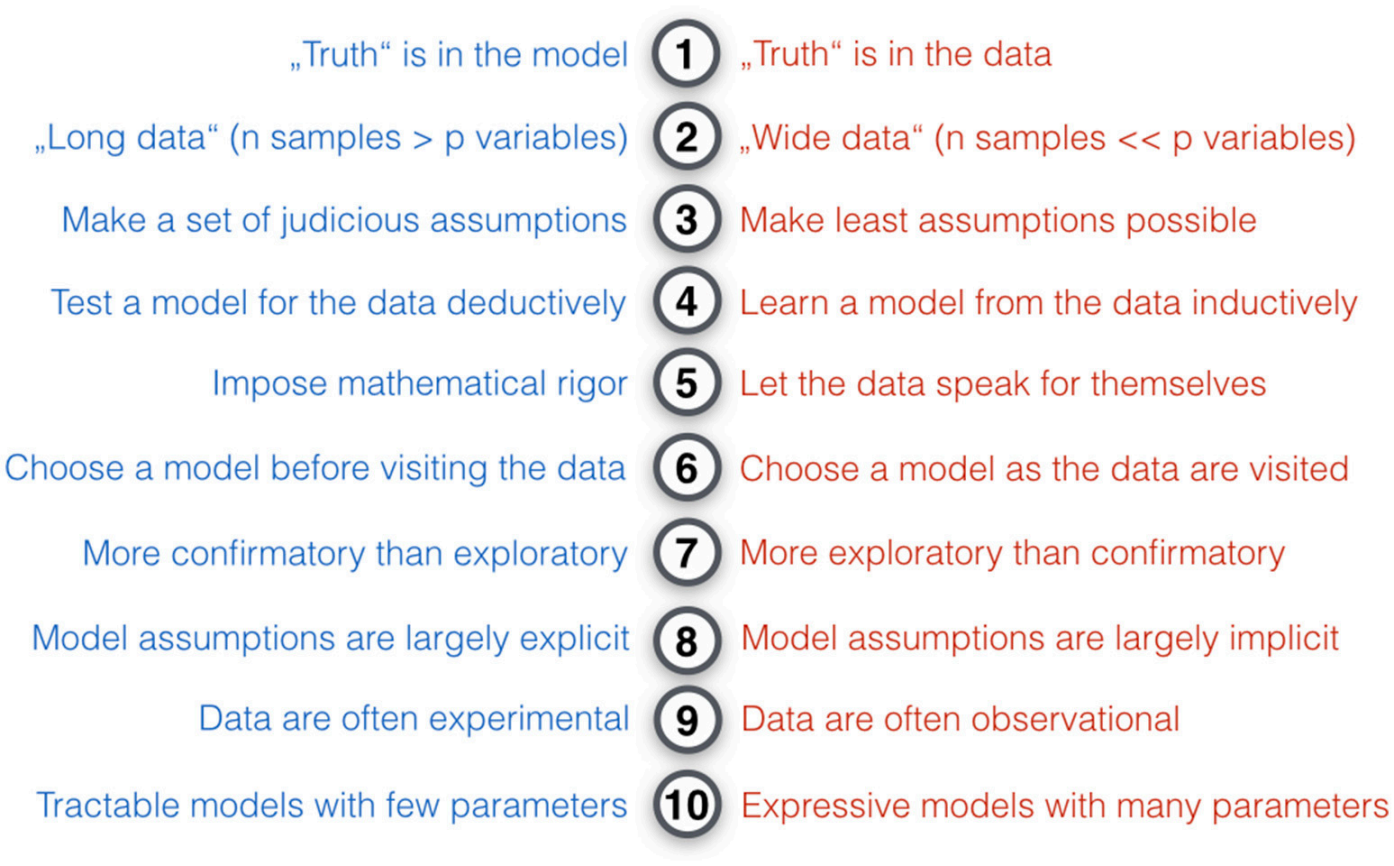
Key differences in the modeling philosophy of classical statistics and statistical learning. Ten modeling intuitions that tend to be relatively more characteristic for classical statistical methods (blue) or pattern-learning methods (red). In comparison to ClSt, StLe “is essentially a form of applied statistics with increased emphasis on the use of computers to statistically estimate complicated functions and a decreased emphasis on proving confidence intervals around these functions” (Goodfellow et al., 2016). Broadly, ClSt tends to be more analytical by imposing mathematical rigor on the phenomenon, whereas StLe tends to be more heuristic by finding useful approximations. In practice, ClSt is probably more often applied to experimental data, where a set of target variables are systematically controlled by the investigator and the brain system under studied has been subject to experimental perturbation. Instead, StLe is probably more often applied to observational data without such structured influence and where the studied system has been left unperturbed. ClSt fully specifies the statistical model at the beginning of the investigation, whereas in StLe there is a bigger emphasis on models that can flexibly adapt to the data (e.g., learning algorithms creating decision trees).

From a conceptual viewpoint (Figure 3), a large majority of statistical methods can be situated somewhere on a continuum between the two poles of ClSt and StLe (Committee on the Analysis of Massive Data et al., 2013; Efron and Hastie, 2016; p. 61). ClSt was mostly fashioned for problems with small samples that can be grasped by plausible models with a small number of parameters chosen by the investigator in an analytical fashion. StLe was mostly fashioned for problems with many variables in potentially large samples with little knowledge of the data-generating process that gets emulated by a mathematical function derived from data in a heuristic fashion. Tools from ClSt therefore typically assume that the data behave according to certain known mechanisms, whereas StLe exploits algorithmic techniques to avoid many a-priori specifications of data-generating mechanisms. Neither ClSt or StLe nor any of the other categories of statistical models can be considered generally superior. This relativism is captured by the so-called *no free lunch theorem*^4^ (Wolpert, 1996): no single statistical strategy can consistently do better in all circumstances (cf. Gigerenzer, 2004). As a very general rule of thumb, ClSt preassumes and formally tests *a model for the data*, whereas StLe extracts and empirically evaluates *a model from the data*.

## Case study one: cognitive contrast analysis and decoding mental states

Vignette: A neuroimaging investigator wants to reveal the neural correlates underlying face processing in humans. 40 healthy, right-handed adults are recruited and undergo a block design experiment run in a 3T MRI scanner with whole-brain coverage. In a passive viewing paradigm, 60 colored stimuli of unfamiliar faces are presented, which have forward head and gaze position. The control condition presents colored pictures of 60 different houses to the participants. In the experimental paradigm, a picture of a face or a house is presented for 2 s in each trial and the interval between trials within each block is randomly jittered varying from 2 to 7 s. The picture stimuli are presented in pseudo-randomized fashion and are counterbalanced in each passively watching participant. Despite the blocked presentation of stimuli, each experiment trial is modeled separately. The fMRI data are analyzed using a GLM as implemented in the SPM software package. Two task regressors are included in the model for the face and house conditions based on the stimulus onsets and viewing durations and using a canonical hemodynamic response function. In the GLM design matrix, the face column and house column are hence set to 1 for brain scans from the corresponding task condition and set to 0 otherwise. Separately in each brain voxel, the GLM parameters are estimated, which fits beta_face_ and beta_house_ regression coefficients to explain the contribution of each experimental task to the neural activity increases and decreases observed in that voxel. A *t*-test can then formally assess whether the fMRI signal in the current voxel is significantly more involved in viewing faces as opposed to the house control condition.

Question: What is the statistical difference between *subtracting* the neural activity from the face vs. house conditions and *decoding* the neural activity during face vs. house processing?

Computing cognitive contrasts is a ClSt approach that was and still is routinely performed in the *mass-univariate regime:* it fits a separate GLM model for each individual voxel in the brain scans and then tests for significant differences between the obtained condition coefficients (Friston et al., 1994). Instead, decoding cognitive processes from neural activity is a StLe approach that is typically performed in a *multivariate regime*: a learning algorithm is trained on a large number of voxel observations in brain scans and then the model's prediction accuracy is evaluated on sets of new brain scans. These ClSt and StLe approaches to identifying the neural correlates underlying cognitive processes of interest are closely related to the notions of *encoding models* and *decoding models*, respectively (Kriegeskorte, 2011; Naselaris et al., 2011; Pedregosa et al., 2015; but see Güçlü and van Gerven, 2015).

Encoding models regress the brain data against a design matrix with indicators of the face vs. house condition and formally test whether the difference is statistically significant. Decoding models typically aim to predict these indicators by training and empirically evaluating classification algorithms on different splits from the whole dataset. In ClSt parlance, the model *explains* the neural activity, the *dependent or explained variable*, measured in each separate brain voxel, by the *beta coefficients* according to the experimental condition indicators in the *design matrix* columns, the *independent or explanatory variables*. That is, the GLM can be used to explain neural activity changes by a linear combination of experimental variables (Naselaris et al., 2011). Answering the same neuroscientific question with decoding models in StLe jargon, the *model weights* of a *classifier* are fitted on the *training set* of the *input data* to *predict* the *class labels*, the *target variables*, and are subsequently evaluated on the *test set* by *cross-validation* to obtain their *out-of-sample generalization performance*. Here, classification algorithms are used to predict entries of the design matrix by identifying a linear or more complicated combination between the many simultaneously considered brain voxels (Pereira et al., 2009). More broadly, ClSt applications in functional neuroimaging tend to estimate the location of cognitive processes from neural activity, whereas many StLe applications estimate properties of neural activity underlying different cognitive tasks.

A key difference between many ClSt-mediated encoding models and StLe-mediated decoding models thus pertains to the direction of statistical estimation between brain space and behavior space (Friston et al., 2008; Varoquaux and Thirion, 2014). It was noted (Friston et al., 2008) that the direction of brain-behavior association is related to the question whether the stimulus indicators in the model act as causes by representing deterministic experimental variables of an encoding model or consequences by representing probabilistic outputs of a decoding model. Such considerations also reveal the intimate relationship of ClSt models to the notion of *forward inference*, while StLe methods are probably more often used for formal *reverse inference* in functional neuroimaging (Poldrack, 2006; Eickhoff et al., 2011; Yarkoni et al., 2011; Varoquaux and Thirion, 2014). On the one hand, *forward inference* relates to encoding models by testing the probability of observing activity in a brain location given knowledge of a psychological process. On the other hand, *reverse inference* relates to brain decoding to the extent that classification algorithms can learn to distinguish experimental fMRI data to belong to two psychological conditions and subsequently be used to estimate the presence of specific cognitive processes based on new neural activity observations (cf. Poldrack, 2006). Finally, establishing a brain-behavior association has been argued to be more important than the actual direction of the mapping function (Friston, 2009). This author stated that “showing that one can decode activity in the visual cortex to classify […] a subject's percept is exactly the same as demonstrating significant visual cortex responses to perceptual changes” and, conversely, “all demonstrations of functionally specialized responses represent an implicit mindreading.”

Conceptually, GLM-based encoding models follow a *localization agenda* by testing hypotheses on *regional effects of functional specialization* in the brain (where?). A *t*-test is used to compare pairs of neural activity estimates to statistically distinguish the target face and the non-target house condition (Friston et al., 1996). Essentially, this test for significant differences between the fitted beta coefficients corresponds to two stimulus indicators based on well-founded arguments from cognitive theory. This statistical approach assumes that *cognitive subtraction* is possible, that is, the regional brain responses of interest can be isolated by contrasting two sets of brain scans that are believed to differ in the cognitive facet of interest (Friston et al., 1996; Stark and Squire, 2001). For one voxel location at a time, an attempt is made to reject the null hypothesis of no difference between the averaged *neural activity level* of a target brain state and the averaged neural activity of a control brain state. It is important to appreciate that the localization agenda thus emphasizes the *relative difference* in fMRI signal during tasks and may neglect the individual neural activity information of each particular task (Logothetis et al., 2001). Note that the univariate GLM analysis can be extended to more than one output (dependent or explained) variable within the ClSt regime by performing a multivariate analysis of covariance (MANCOVA). This allows for tests of more complex hypotheses but incurs multivariate normality assumptions (Kriegeskorte, 2011).

More generally, it is seldom mentioned that the standard GLM would not have been solvable for unique solutions in the high-dimensional “*n* < < *p*” regime, instead of fitting one model for each voxel in the brain scans. This is because the number of brain voxels *p* exceed by far the number of data samples n (i.e., leading to an under-determined system of equations), which incapacitates many statistical estimators from ClSt (cf. Giraud, 2014; Hastie et al., 2015). Regularization by sparsity-inducing norms, such as in modern *penalized* regression analysis using the LASSO and ElasticNet, emerged only later (Tibshirani, 1996; Zou and Hastie, 2005) as a principled StLe strategy to de-escalate the need for dimensionality reduction or preliminary filtering of important voxels and to enable the tractability of the high-dimensional analysis setting.

Because hypothesis testing for significant differences between beta coefficients of fitted GLMs relies on comparing the means of neural activity measurements, the results from statistical tests are not corrupted by the conventionally applied spatial smoothing with a Gaussian filter. On the contrary, this image preprocessing step even helps the correction for multiple comparisons based on random fields theory (cf. below), alleviates inter-individual neuroanatomical variability, and can thus increases sensitivity. Spatial smoothing however discards fine-grained neural activity patterns spatially distributed across voxels that potentially carry information associated with mental operations (cf. Kamitani and Sawahata, 2010; Haynes, 2015). Indeed, some authors believe that sensory, cognitive, and motor processes manifest themselves as “neuronal population codes” (Averbeck et al., 2006). Relevance of such population codes in human neuroimaging was for instance suggested by revealing subject-specific neural responses in the fusiform gyrus to facial stimuli (Saygin et al., 2012). In applications of StLe models, the spatial smoothing step is therefore often skipped because the “decoding” algorithms precisely exploit the locally varying structure of the salt-and-pepper patterns in fMRI signals.

In so doing, decoding models use learning algorithms in an *information agenda* by showing *generalization of robust patterns* to new brain activity acquisitions (Kriegeskorte et al., 2006; Mur et al., 2009; de-Wit et al., 2016). Information that is weak in one voxel but spatially distributed across voxels can be effectively harvested in a structure-preserving fashion (Haynes and Rees, 2006; Haynes, 2015). This modeling agenda is focused on the whole *neural activity pattern*, in contrast to the localization agenda dedicated to separate increases or decreases in *neural activity level*. For instance, the default mode network typically exhibits activity *decreases* at the onset of many psychological tasks with visual or other sensory stimuli, whereas the induced activity *patterns* in that less activated network may nevertheless functionally subserve task execution (Bzdok et al., 2016; Christoff et al., 2016). Some brain-behavior associations might only emerge when simultaneously capturing neural activity in a group of voxels but disappear in single-voxel approaches, such as mass-univariate GLM analyses (cf. Davatzikos, 2004). Note that, analogous to multivariate variants of the GLM, decoding could also be replaced by classical statistical approaches (cf. Haxby et al., 2001; Brodersen et al., 2011a). For many linear classification algorithm trained to predict face vs. house stimuli based on many brain voxels, model fitting typically searches iteratively through the *hypothesis space* (= *function space*) of the chosen learning model. In our case, the final hypothesis selected by the linear classifier commonly corresponds to one specific combination of model weights (i.e., a weighted contribution of individual brain measurements) that equates with one mapping function from the neural activity features to the face vs. house target variable.

Among other views, it has previously been proposed (Brodersen, 2009) that four types of neuroscientific questions become readily quantifiable through StLe applications to neuroimaging: (i) *Where* is an information category neurally processed? This can extend the interpretational spectrum from increase and decrease of neural activity to the existence of complex combinations of activity variations distributed across voxels. For instance, across-voxel linear correlation could decode object categories from the ventral temporal cortex even after excluding the fusiform gyrus, which is known to be responsive to object stimuli (Haxby et al., 2001). (ii) *Whether* a given information category is reflected by neural activity? This can extend the interpretational spectrum to topographically similar but neurally distinct processes that potentially underlie different cognitive facets. For instance, linear classifiers could successfully distinguish whether a subject is attending to the first or second of two simultaneously presented stimuli (Kamitani and Tong, 2005). (iii) *When* is an information category generated (i.e., onset), processed (i.e., duration), and bound (i.e., alteration)? When applying classifiers to neural time series, the interpretational spectrum can be extended to the beginning, evolution, and end of distinct cognitive facets. For instance, different classifiers have been demonstrated to map the decodability time structure of mental operation sequences (King and Dehaene, 2014). (iv) More controversially, *how* is an information category neurally processed? The interpretational spectrum can be extended to computational properties of the neural processes, including processing in brain regions vs. brain networks or isolated vs. partially shared processing facets. For instance, a classifier trained for evolutionarily conserved eye gaze movement was able to decode evolutionarily more recent mathematical calculation processes as a possible case of “neural recycling” in the human brain (Knops et al., 2009; Anderson, 2010). As an important caveat in interpreting StLe models, the particular technical properties of a chosen learning algorithm (e.g., linear vs. non-linear support vector machines) can probably seldom serve as a convincing argument for reverse-engineering mechanisms of neural information processing as measured by fMRI scanning (cf. Misaki et al., 2010).

In sum, the statistical properties of ClSt and StLe methods have characteristic consequences in neuroimaging analysis and interpretation. They can hence offer different access routes and complementary answers to identical neuroscientific questions.

## Case study two: small volume correction and searchlight analysis

Vignette: The neuroimaging experiment from case study 1 successfully identified the fusiform gyrus of the ventral visual stream to be more responsive to face stimuli than house stimuli. However, the investigator's initial hypothesis of also observing face-responsive neural activity in the ventromedial prefrontal cortex could not be confirmed in the *whole-brain* analyses. The investigator therefore wants to follow up with a *topographically focused* approach that examines differences in neural activity between the face and house conditions exclusively in the ventromedial prefrontal cortex.

Question: What are the statistical implications of delineating task-relevant neural responses in a spatially constrained search space rather than analyzing brain measurements of the entire brain?

A popular ClSt approach to corroborate less pronounced neural activity findings is *small volume correction*. This region of interest (ROI) analysis involves application of the mass-univariate GLM approach only to the ventromedial prefrontal cortex as a preselected biological compartment, rather than considering the gray-matter voxels of the entire brain in a naïve, topographically unconstrained fashion. Small volume correction allows for significant findings in the ROI that remain sub-threshold after accounting for the tens of thousands of multiple comparisons in the whole-brain GLM analysis. Small volume correction is therefore a simple means to alleviate the multiple-comparisons problem that motivated more than two decades of still ongoing methodological developments in the neuroimaging domain (Worsley et al., 1992; Smith et al., 2001; Friston, 2006; Nichols, 2012). Whole-brain GLM results were initially reported as uncorrected findings without accounting for multiple comparisons, then with Bonferroni's family wise error (FWE) correction, later by random field theory correction using neural activity height (or clusters), followed by false discovery rate (FDR) (Genovese et al., 2002) and slowly increasing adoption of cluster-thresholding for voxel-level inference via permutation testing (Smith and Nichols, 2009). Rather than the isolated voxel, it has early been discussed that a possibly better unit of interest should be spatially neighboring voxel groups (see here for an overview: Chumbley and Friston, 2009). The setting of high regional correlation of neural activity was successfully addressed by random field theory that provide inferences not about individual voxels but topological features in the underlying (spatially continuous) effects. This topological inference is used to identify clusters of relevant neural activity changes from their peak, size, or mass (Worsley et al., 1992). Importantly, the spatial dependencies of voxel observations were not incorporated into the GLM estimation step, but instead taken into account during the subsequent model inference step to alleviate the multiple-comparisons problem.

A related cousin of small volume correction in the StLe world would be to apply classification algorithms to a subset of voxels to be considered as input to the model (i.e., *feature selection*). In particular, *searchlight analysis* is an increasingly popular learning technique that can identify *locally constrained multivariate patterns* in neural activity (Friman et al., 2001; Kriegeskorte et al., 2006). For each voxel in the ventromedial prefrontal cortex, the brain measurements of the immediate neighborhood are first collected (e.g., radius of 10 mm voxels). In each such searchlight, a classification algorithm, for instance linear support vector machines, is then trained on one part of the brain scans (*training set*) and subsequently applied to determine the prediction accuracy in the remaining, unseen brain scans (*test set*). In this StLe approach, the excess of brain voxels is handled by performing pattern recognition analysis in only dozens of locally adjacent voxel neighborhoods at a time. Finally, the mean classification accuracy of face vs. house stimuli across all permutations over the brain data is mapped to the center of each considered sphere. The searchlight is then moved through the ROI until each seed voxel had once been the center voxel of the searchlight. This yields a voxel-wise classification map of accuracy estimates for the entire ventromedial prefrontal cortex. Consistent with the information agenda (cf. above), searchlight analysis quantifies the extent to which (local) neural activity *patterns* can *predict* the difference between the house and face conditions. It contrasts small volume correction that determines whether one experimental condition exhibited a significant neural activity *increase* or *decrease* relative to a particular other experimental condition, consistent with the localization agenda. Further, searchlight analysis alleviates the burden of abundant input variables by fitting learning algorithms restricted to the voxels in small sphere neighborhoods. However, the searchlight procedure thus yields many prediction performances for many brain locations, which motivates correction for multiple comparisons across the considered neighborhoods.

When considering high-dimensional brain scans through the ClSt lens, the statistical challenge resides in solving the *multiple-comparisons problem* (Nichols and Hayasaka, 2003; Nichols, 2012). From the StLe stance, however, it is the *curse of dimensionality* and *overfitting* that statistical analyses need to tackle (Friston et al., 2008; Domingos, 2012). Many neuroimaging analyses based on ClSt methods can be viewed as testing a particular hypothesis (i.e., the null hypothesis) repeatedly in a large number of separate voxels. In contrast, testing whether learning algorithm extrapolate to new brain data can be viewed as searching through thousands of different hypotheses in a single process (i.e., walking through the hypothesis space; cf. above) (Shalev-Shwartz and Ben-David, 2014).

As common brain scans offer measurements of >100,000 brain locations, a mass-univariate GLM analysis typically entails the same statistical test to be applied >100,000 times. The more often the investigator tests a hypothesis of relevance for a brain location, the more locations will be falsely detected as relevant (false positive, Type I error), especially in the noisy neuroimaging data. All dimensions in the brain data (i.e., voxel variables) are implicitly treated as equally important and no neighborhoods of most expected variation are statistically exploited (Hastie et al., 2001). Hence, the absence of restrictions on observable structure in the set of data variables during the statistical modeling of neuroimaging data takes a heavy toll at the final inference step. This is where *random field theory* comes to the rescue. As noted above, this form of topological inference dispenses with the problem of inferring which voxels are significant and tries to identify significant topological features in the underlying distributed responses. By definition, topological features like maxima are sparse events and can be thought of as a form of dimensionality reduction—not in data space but in the statistical characterization of where neural responses occur.

This is contrasted by the high-dimensional StLe regime, where the initial model family chosen by the investigator determines the complexity restrictions to all data dimensions (i.e., all voxels, not single voxels) that are imposed explicitly or implicitly by the model structure. Model choice predisposes existing but unknown low-dimensional neighborhoods in the full voxel space to achieve the prediction task. Here, the toll is taken at the beginning of the investigation because there are so many different alternative model choices that would impose a different set of complexity constraints to the high-dimensional measurements in the brain. For instance, signals from “brain regions” are likely to be well approximated by models that impose discrete, locally constant compartments on the data (e.g., *k*-means or spatially constrained Ward clustering). Instead, tuning model choice to signals from macroscopical “brain networks” should impose overlapping, locally continuous data compartments (e.g., independent component analysis or sparse principal component analysis) (Yeo et al., 2014; Bzdok and Yeo, 2017; Bzdok et al., 2017).

Exploiting such *effective dimensions* in the neuroimaging data (i.e., coherent brain-behavior associations involving many distributed brain voxels) is a rare opportunity to simultaneously reduce the *model bias* and *model variance*, despite their typical inverse relationship (Hastie et al., 2001). Model bias relates to prediction failures incurred because the learning algorithm can systematically not represent certain parts of the underlying relationship between brain scans and experimental conditions (formally, the deviation between the target function and the average function space of the model). Model variance relates to prediction failures incurred by noise in the estimation of the optimal brain-behavior association (formally, the difference between the best-choice input-output relation and the average function space of the model). A model that is too simple to capture a brain-behavior association probably underfits due to high bias. Yet, an overly complex model probably overfits due to high variance. Generally, high-variance approaches are better at *approximating* the “true” brain-behavior relation (i.e., in-sample model estimation), while high-bias approaches have a higher chance of *generalizing* the identified pattern to new observations (i.e., out-of-sample model evaluation). The bias-variance tradeoff can be useful in explaining why applications of statistical models intimately depend on (i) the amount of available data, (ii) the typically not known amount of noise in the data, and (iii) the unknown complexity of the target function in nature (Abu-Mostafa et al., 2012).

Learning algorithms that overcome the curse of dimensionality—extracting coherent patterns from all considered brain voxels at once—typically incorporate an implicit bias for anisotropic neighborhoods in the data (Hastie et al., 2001; Bach, 2014; Bzdok et al., 2015). Put differently, prediction models successful in the high-dimensional setting have an in-built specialization to representing types of functions that are compatible with the structure to be uncovered in the brain data. Knowledge embodied in a learning algorithm suited to a particular application domain can better calibrate the sweet spot between underfitting and overfitting. When applying a model without any complexity restrictions to high-dimensional data generalization becomes difficult to impossible because all directions in the data (i.e., individual brain voxels) are treated equally with isotropic structure. At the root of the problem, all data samples look virtually identical to the learning algorithm in high-dimensional data scenarios (Bellman, 1961). The learning algorithm will struggle to see through the idiosyncracies in the data, will tend to overfit, and thus be unlikely to generalize to new observations. Such considerations provide insight into why the multiple-comparisons problem is more often an issue in encoding studies, while overfitting is more closely related to decoding studies (Friston et al., 2008). The juxtaposition of ClSt and StLe views offers insights into why restricting neural data analysis to an ROI with fewer voxels, rather than the whole brain, simultaneously alleviates both the multiple-comparisons problem (ClSt) and the curse of dimensionality (StLe).

As an practical summary, drawing classical inference in neuroimaging data has largely been performed by considering each voxel independently and by massive simultaneous testing of a same null hypothesis in all observed voxels. This has incurred a multiple-comparisons problem difficult enough that common approaches may still be prone to incorrect results (Efron, 2012). In contrast, aiming for generalization of a pattern in high-dimensional neuroimaging data to new observations in the brain incurs the equally challenging curse of dimensionality. Successfully accounting for the high number of input dimensions will probably depend on learning models that impose neurobiologically justified bias and keeping the variance under control by dimensionality reduction and regularization techniques.

**Figure 4:**
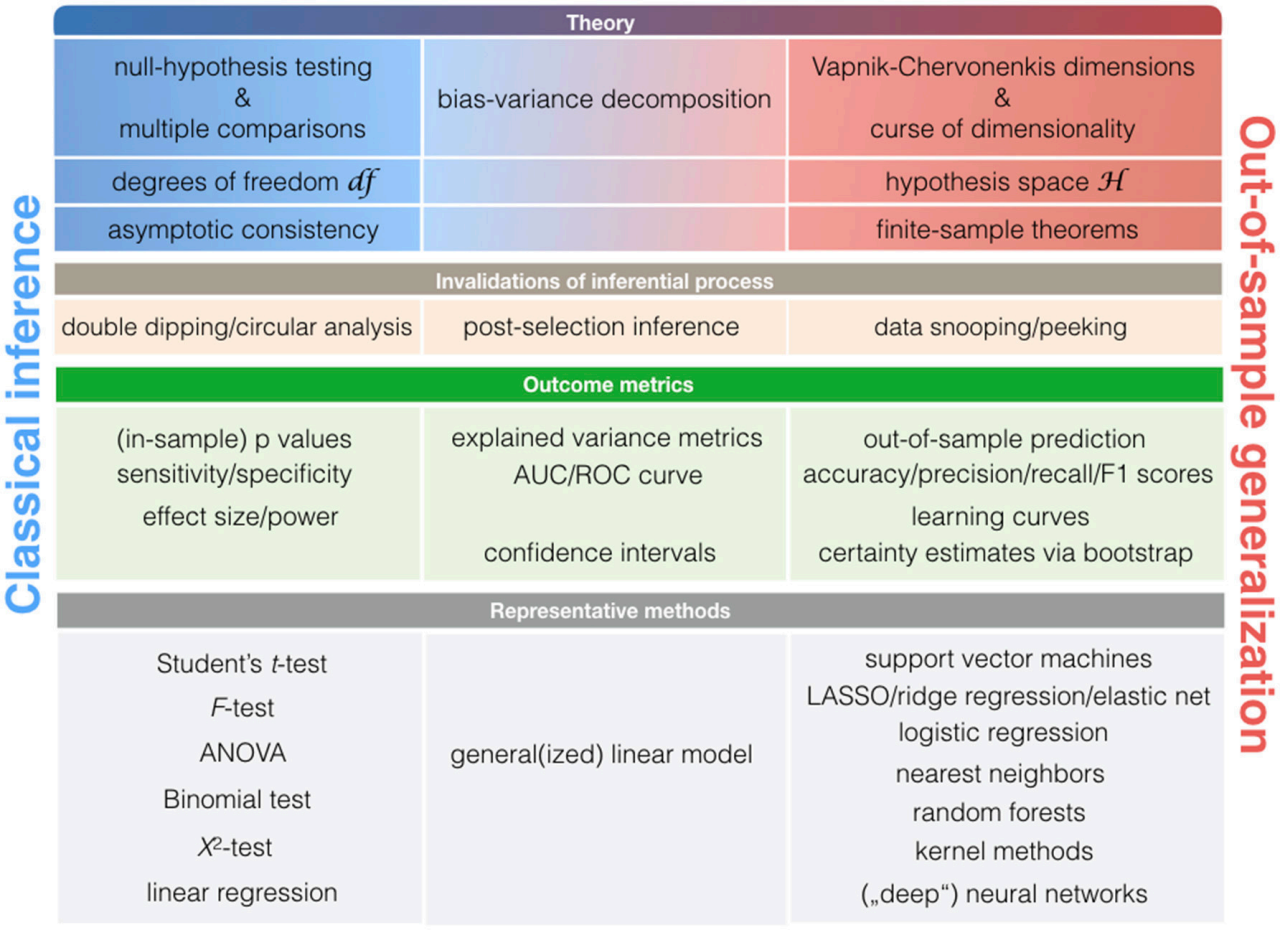
Key concepts in classical statistics and statistical learning. Schematic with statistical notions that are relatively more associated with classical statistical methods (left column) or pattern-learning methods (right column). As there is a smooth transition between the classical statistical toolkit and learning algorithms, some notions may be closely associated with both statistical cultures (middle column).

More broadly, asking at what point new neurobiological knowledge is arising during ClSt and StLe investigations relies on largely distinct theoretical frameworks that revolve around *null-hypothesis testing* and *statistical learning theory* (Figure 4). Both ClSt and StLe methods share the common goal of demonstrating relevance of a given effect in the data beyond the sample brain scans at hand. However, the attempt to show successful extrapolation of a statistical relationship at the general population is embedded in different mathematical contexts. Knowledge generation in ClSt and StLe is hence rooted in different notions of statistical inference.

ClSt laid down its most important inferential framework in the Popperian spirit of critical empiricism (Popper, 1935/2005): scientific progress is to be made by continuous replacement of current hypotheses by ever more pertinent hypotheses using *falsification*. The rationale behind hypothesis falsification is that one counterexample can reject a theory by *deductive reasoning*, while any quantity of evidence can not confirm a given theory by inductive reasoning (Goodman, 1999). The investigator verbalizes two mutually exclusive hypotheses by domain-informed judgment. The *alternative hypothesis* should be conceived as the outcome intended by the investigator and to contradict the state of the art of the research topic. The *null hypothesis* represents the devil's advocate argument that the investigator wants to reject (i.e., falsify) and it should automatically deduce from the newly articulated alternative hypothesis. A conventional 5%-threshold (i.e., equating with roughly two standard deviations) guards against rejection due to the idiosyncrasies of the sample that are not representative of the general population. If the data have a probability of ≤5% given the null hypothesis [P(result|H_0_)], it is evaluated to be significant. Such a *test for statistical significance* indicates a difference between two means with a 5% chance of being a false positive finding. If the null hypothesis can not be rejected (which depends on power), then the test yields no conclusive result, rather than a null result (Schmidt, 1996). In this way, classical hypothesis testing continuously replaces currently embraced hypotheses explaining a phenomenon in nature by better hypotheses with more empirical support in a Darwinian selection process. Finally, Fisher, Neyman, and Pearson intended hypothesis testing as a marker for further investigation, rather than an off-the-shelf decision-making instrument (Cohen, 1994; Nuzzo, 2014).

In StLe instead, answers to how neurobiological conclusions can be drawn from a dataset at hand are provided by the *Vapnik-Chervonenkis dimensions* (VC dimensions) from *statistical learning theory* (Vapnik, 1989, 1996). The VC dimensions of a pattern-learning algorithm quantify the probability at which the distinction between the neural correlates underlying the face vs. house conditions can be captured and used for correct predictions in new, possibly later acquired brain scans from the same cognitive experiment (i.e., *out-of-sample generalization*). Such statistical approaches implement the *inductive* strategy to learn general principles (i.e., the neural signature associated with given cognitive processes) from a series of exemplary brain measurements, which contrasts the *deductive* strategy of rejecting a certain null hypothesis based on counterexamples (cf. Tenenbaum et al., 2011; Bengio, 2014; Lake et al., 2015). The VC dimensions measure how complicated the examined relationship between brain scans and experimental conditions could become—in other words, the richness of the representation which can be instantiated by the used model, the complexity capacity of its *hypothesis space*, the “wiggliness” of the decision boundary used to distinguish examples from several classes, or, more intuitively, the “currency” of learnability. VC dimensions are derived from the maximal number of different brain scans that can be correctly detected to belong to either the house condition or the face condition by a given model. The VC dimensions thus provide a theoretical guideline for the largest set of brain scan examples fed into a learning algorithm such that this model is able to guarantee zero classification errors.

As one of the most important results from statistical learning theory, in any intelligent learning system, the opportunity to derive abstract patterns in the world by reducing the discrepancy between prediction error from training data (in-sample estimate) and prediction error from independent test data (out-of-sample estimate) decreases with the higher model capacity and increases with the number of available training observations (Vapnik and Kotz, 1982; Vapnik, 1996). In brain imaging, a learning algorithm is hence theoretically backed up to successfully predict outcomes in future brain scans with high probability if the choosen model ignores structure that is overly complicated, such as higher-order non-linearities between many brain voxels, and if the model is provided with a sufficient number of training brain scans. Hence, VC dimensions provide explanations why increasing the number of considered brain voxels as input features (i.e., entailing increased number of model parameters) or using a more sophisticated prediction model, requires more training data for successful generalization. Notably, the VC dimensions (analogous to null-hypothesis testing) are unrelated to the *target function*, as the “true” mechanisms underlying the studied phenomenon in nature. Nevertheless, the VC dimensions provide justification that a certain learning model can be used to approximate that target function by fitting a model to a collection of input-output pairs. In short, VC dimensions is among the best frameworks to derive theoretical errors bounds for predictive models (Abu-Mostafa et al., 2012).

Further, some common invalidations of the ClSt and StLe statistical concern in neuroimaging studies performing classical inference is *double dipping* or *circular analysis* (Kriegeskorte et al., 2009). This occurs when, for instance, first correlating a behavioral measure with brain activity and then using the identified subset of brain voxels for a second correlation analysis with that same behavioral measurement (Lieberman et al., 2009; Vul et al., 2009). In this scenario, voxels are submitted to two statistical tests with the same goal in a nested, non-independent fashion^5^ (Freedman, 1983). This corrupts the *validity of the null hypothesis* on which the reported test results conditionally depend. Importantly, this case of repeating a same statistical estimation with iteratively pruned data selections (on the training data split) is a valid routine in the StLe framework, such as in recursive feature extraction (Guyon et al., 2002; Hanson and Halchenko, 2008). However, double-dipping or circular analysis in ClSt applications to neuroimaging data have an analog in StLe analyses aiming at out-of-sample generalization: *data-snooping* or *peeking* (Pereira et al., 2009; Abu-Mostafa et al., 2012; Fithian et al., 2014). This can occur, for instance, when performing simple (e.g., mean-centering) or more involved (e.g., *k*-means clustering) target-variable-dependent or -independent preprocessing on the entire dataset if it should be applied separately to the training sets and test sets. Data-snooping can lead to overly optimistic cross-validation estimates and a trained learning algorithm that fails on fresh data drawn from the same distribution (Abu-Mostafa et al., 2012). Rather than a corrupted null hypothesis, it is the *error bounds of the VC dimensions that are loosened* and, ultimately, invalidated because information from the concealed test set influences model selection on the training set.

In sum, statistical inference in ClSt is drawn by using the *entire data* at hand to *formally test* for *theoretically guaranteed* extrapolation of an effect to the general population. In stark contrast, inferential conclusions in StLe are typically drawn by fitting a model on a *larger part of the data* at hand (i.e., in-sample model selection) and *empirically testing* for successful extrapolation to an independent, smaller part of the data (i.e., out-of-sample model evaluation). As such, ClSt has a focus on *in-sample estimates* and *explained-variance* metrics that measure some form of goodness of fit, while StLe has a focus on *out-of-sample estimates* and *prediction accuracy*.

## Case study three: significant group differences and predicting the group of participants

Vignette: After isolating the neural correlates underlying face processing, the neuroimaging investigator wants to examine their relevance in psychiatric disease. In addition to the 40 healthy participants, 40 patients diagnosed with schizophrenia are recruited and administered the same experimental paradigm and set of face and house pictures. In this clinical fMRI study on group differences, the investigator wants to explore possible imaging-derived markers that index deficits in social-affective processing in patients carrying a diagnosis of schizophrenia.

Question: Can metrics of statistical relevance from ClSt and StLe be combined to corroborate a given candidate biomarker?

**Figure 5:**
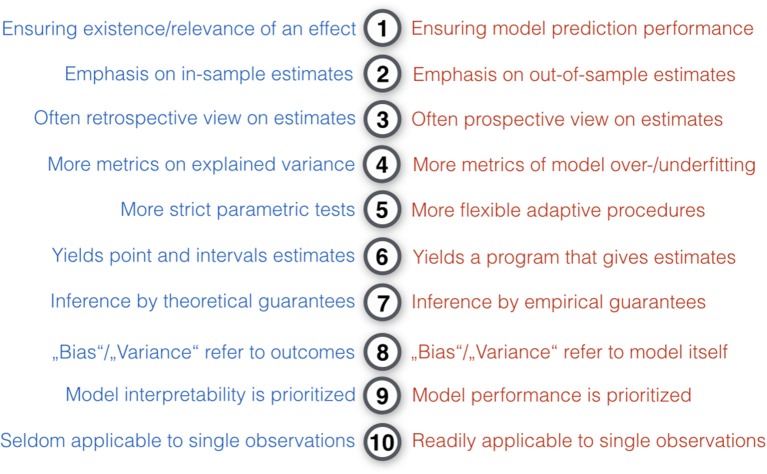
Key differences between measuring outcomes in classical statistics and statistical learning. Ten intuitions on quantifying statistical modeling outcomes that tend to be relatively more true for classical statistical methods (blue) or pattern-learning methods (red). ClSt typically yields point estimates and interval estimates (e.g., *p*-values, variances, confidence intervals), whereas StLe frequently outputs a function or a program that can yield point and interval estimates on new observations (e.g., the *k*-means centroids or a trained classifier's decision function can be applied to new data). In many cases, classical inference is a judgment about an entire data sample, whereas a trained predictive model can obtain quantitative answers from a single data point.

Many investigators in imaging neuroscience share a background in psychology, biology, or medicine, which includes training in traditional “textbook” statistics. Many neuroscientists have thus adopted a natural habit of assessing the quality of statistical relationships by means of *p*-values, effect sizes, confidence intervals, and statistical power. These are ubiquitously taught and used at many universities, although they are not the only coherent set of statistical diagnostics (Figure 5). These outcome metrics from ClSt may for instance be less familiar to some scientists with a background in computer science, physics, engineering, or philosophy. As an equally legitimate and internally coherent, yet less widely known diagnostic toolkit from the StLe community, prediction accuracy, precision, recall, confusion matrices, F1 score, and learning curves can also be used to measure the relevance of statistical relationships (Abu-Mostafa et al., 2012; Yarkoni and Westfall, 2017).

On a general basis, applications of ClSt and StLe methods may not judge findings on identical grounds (Breiman, 2001; Shmueli, 2010; Lo et al., 2015). There is an often-overlooked misconception that models with high explanatory performance do necessarily exhibit high predictive performance (Wu et al., 2009; Lo et al., 2015; Yarkoni and Westfall, 2017). For instance, brain voxels in ventral visual stream found to well *explain* the difference between face processing in healthy and schizophrenic participants based on an ANOVA may not in all cases be the best brain features to train a support vector machine to *predict* this group effect in new participants. An important outcome measure in ClSt is the quantified *significance* associated with a statistical relationship between few variables given a pre-specified model. ClSt tends to *test for a particular structure* in the brain data based on *analytical guarantees*, in form of as mathematical convergence theorems about approximating the population properties with increasing sample size. The outcome measure for StLe is the quantified *generalization of patterns* between many variables or, more generally, the robustness of special structure in the data (Hastie et al., 2001). In the neuroimaging literature, reports of statistical outcomes have previously been noted to confuse diagnostic measures from classical statistics and statistical learning (Friston, 2012).

For neuroscientists adopting a ClSt culture computing *p*-values takes a central position. The *p-value* denotes the probability of observing a result at least as extreme as a test statistic, assuming the null hypothesis is true. Results are considered significant when it is equal or below a pre-specified value, like *p* = 0.05 (Anderson et al., 2000). Under the condition of sufficiently high power (cf. below), it quantifies the strength of evidence against the null hypothesis as a continuous function (Rosnow and Rosenthal, 1989). Counterintuitively, it is not an immediate judgment on the alternative hypothesis H_1_ preferred by the investigator (Cohen, 1994; Anderson et al., 2000). *P*-values do also not qualify the possibility of replication. It is another important caveat that a finding in the brain becomes more statistically significant (i.e., lower *p*-value) with increasing sample size (Berkson, 1938; Miller et al., 2016).

The essentially binary *p*-value (i.e., significant vs. not significant) is therefore often complemented by continuous *effect size* measures for the importance of rejecting H_0_. The effect size allows the identification of marginal effects that pass the statistical significance threshold but are not practically relevant in the real world. The *p*-value is a deductive *inferential* measure, whereas the effect size is a *descriptive* measure that follows neither inductive nor deductive reasoning. The (normalized) effect size can be viewed as the strength of a statistical relationship—how much H_0_ deviates from H_1_, or the likely presence of an effect in the general population (Chow, 1998; Ferguson, 2009; Kelley and Preacher, 2012). This diagnostic measure is often unit-free, sample-size independent, and typically standardized. As a property of the actual statistical test, the effect size can be essential to report for biological understanding, but has different names and takes various forms, such as *rho* in Pearson correlation, *eta*^*2*^ in explained variances, and *Cohen's d* in differences between group averages.

Additionally, the certainty of a *point estimate* (i.e., the outcome is a value) can be expressed by an *interval estimate* (i.e., the outcome is a value range) using *confidence intervals* (Casella and Berger, 2002). These variability diagnostics indicate a range of values between which the true value will fall a given proportion of the time (Estes, 1997; Nickerson, 2000; Cumming, 2009). Typically, a 95% confidence interval is spanned around the population mean in 19 out of 20 cases across all observed samples. The tighter the confidence interval, the smaller the variance of the point estimate of the population parameter in each drawn sample. The estimation of confidence intervals is influenced by sample size and population variability. Confidence intervals may be asymmetrical (ignored by Gaussianity assumptions; Efron, 2012), can be reported for different statistics and with different percentage borders. Notably, they can be used as a viable surrogate for formal tests of statistical significance in many scenarios (Cumming, 2009).

Some confidence intervals can be computed in various data scenarios and statistical regimes, whereas the *power* may be especially meaningful within the culture of classical hypothesis testing (Cohen, 1977, 1992; Oakes, 1986). To estimate power the investigator needs to specify the true effect size and variance under H_1_. The ClSt-minded investigator can then estimate the probability for rejecting null hypotheses that should be rejected, at the given threshold alpha and given that H_1_ is true. A high power thus ensures that statistically significant and non-significant tests indeed reflect a property of the population (Chow, 1998). Intuitively, a small confidence interval around a relevant effect suggests high statistical power. False negatives (i.e., Type II errors, beta error) become less likely with higher power (= 1—beta error) (cf. Ioannidis, 2005). Concretely, an underpowered investigation means that the investigator is less likely to be able to distinguish between H_0_ and H_1_ at the specified significance threshold alpha. Power calculations depend on several factors, including significance threshold alpha, the effect size in the population, variation in the population, sample size *n*, and experimental design (Cohen, 1992).

While neuroimaging studies based on classical statistical inference ubiquitously report *p*-values and confidence intervals, there have however been few reports of effect size in the neuroimaging literature (Kriegeskorte et al., 2010). Effect sizes are however necessary to compute power estimates. This explains the even rarer occurrence of power calculations in the neuroimaging literature (Yarkoni and Braver, 2010; but see Poldrack et al., 2017). Given the importance of *p*-values *and* effect sizes, the goal of computing both these useful statistics, such as for group differences in the neural processing of face stimuli, can be achieved based on two independent samples of these experimental data (especially if some selection process has been used). One sample would be used to perform statistical inference on the neural activity change yielding a *p*-value and one sample to obtain unbiased effect sizes. Further, it has been previously emphasized (Friston, 2012) that *p*-values and effect sizes reflect in-sample estimates in a retrospective inference regime (ClSt). These metrics find an analog in out-of-sample estimates issued from cross-validation in a prospective prediction regime (StLe). In-sample effect sizes are typically an *optimistic* estimate of the “true” effect size (inflated by high significance thresholds), whereas out-of-sample effect sizes are *unbiased* estimates of the “true” effect size.

In the high-dimensional scenario, the StLe-minded investigator analyzing “wide” neuroimaging data in our case, computing, and judging statistical significance by *p*-values can become challenging (Bühlmann and Van De Geer, 2011; Efron, 2012; James et al., 2013). Instead, *classification accuracy* on fresh data is a frequently reported performance metric in neuroimaging studies using learning algorithms. The *classification accuracy* is a simple summary statistic that captures the fraction of correct prediction instances among all performed applications of a fitted model. Basing interpretation on accuracy alone can be an insufficient diagnostic because it is frequently influenced by the number of samples, the local characteristics of hemodynamic responses, efficiency of experimental design, data folding into train and test sets, and differences in the feature number *p* (Haynes, 2015). A potentially under-exploited data-driven tool in this context is *bootstrapping*. The archetypical example of computer-intensive statistical method enables population-level inference of unknown distributions largely independent of model complexity by repeated random draws from the neuroimaging data sample at hand (Efron, 1979; Efron and Tibshirani, 1994). This opportunity to equip various point estimates by an interval estimate of certainty (e.g., the possibly asymmetrical interval for the “true” accuracy of a classifier) is unfortunately seldom embraced in neuroimaging today (but see Bellec et al., 2010; Pernet et al., 2011; Vogelstein et al., 2014). Besides providing confidence intervals, bootstrapping can also perform non-parametric null hypothesis testing. This may be one of few examples of a direct connection between ClSt and StLe methodology. Alternatively, *binomial tests* have been used to obtain a *p*-value estimate of statistical significance from accuracies and other performance scores (Pereira et al., 2009; Brodersen et al., 2013; Hanke et al., 2015) in the binary classification setting. It has frequently been employed to reject the null hypothesis that two categories occur equally often. There are however increasing concerns about the validity of this approach if statistical independence between the performance estimates (e.g., prediction accuracies from each cross-validation fold) is in question (Pereira and Botvinick, 2011; Noirhomme et al., 2014; Jamalabadi et al., 2016). Yet another option to derive *p*-values from classification performances of two groups is *label permutation* based on non-parametric resampling procedures (Nichols and Holmes, 2002; Golland and Fischl, 2003). This algorithmic significance-testing tool can serve to reject the null hypothesis that the neuroimaging data do not contain relevant information about the group labels in many complex data analysis settings.

The neuroscientist who adopted a StLe culture is in the habit of corroborating prediction accuracies using *cross-validation:* the de facto standard to obtain an unbiased estimate of a model's capacity to generalize beyond the brain scans at hand (Hastie et al., 2001; Bishop, 2006). *Model assessment* is commonly done by training on a bigger subset of the available data (i.e., *training set* for *in-sample performance*) and subsequent application of the trained model to the typically smaller remaining part of data (i.e., *test set* for *out-of-sample performance*), both assumed to be drawn from the same distribution. Cross-validation typically divides the sample into data splits such that the class label (i.e., healthy vs. schizophrenic) of each data point is to be predicted once. The pairs of model-predicted label and the corresponding true label for each data point (i.e., brain scan) in the dataset can then be submitted to the quality measures (Powers, 2011), including *prediction accuracy* (inversely related to *prediction error*), *precision, recall*, and *F1 score*. Accuracy and the other performance metrics are often computed separately on the training set and the test set. Additionally, the measures from training and testing can be expressed by their inverse (e.g., *training error* as *in-sample error* and *test error* as *out-of-sample error*) because the positive and negative cases are interchangeable.

The classification accuracy can be further decomposed into group-wise metrics based on the so-called *confusion matrix*, the juxtaposition of the true and predicted group memberships. The *precision* measures (Table 1) how many of the labels predicted from brain scans are correct, that is, how many participants predicted to belong to a certain class really belong to that class. Put differently, among the participants predicted to suffer from schizophrenia, how many have really been diagnosed with that disease? On the other hand, the *recall* measures how many labels are correctly predicted, that is, how many members of a class were predicted to really belong to that class. Hence, among the participants known to be affected by schizophrenia, how many were actually detected as such? Precision can be viewed as a measure of “exactness” and recall as a measure of “completeness” (Powers, 2011).

**Table 1:** Metrics used to create ROC curves.

**Notion**	**Formula**
Specificity	true negative/(true negative + false positive)
Sensitivity/Recall	true positive/(true positive + false negative)
Precision	true positive/(true positive + false positive)

Neither accuracy, precision, or recall allow injecting subjective importance into the evaluation process of the learning algorithm. This disadvantage is addressed by the *F*_*beta*_ *score*: a weighted combination of the precision and recall prediction scores. Concretely, the F_1_ score would equally weigh precision and recall of class predictions, while the F_0.5_ score puts more emphasis on precision and the F_2_ score more on recall. Moreover, applications of recall, precision, and F_beta_ scores have been noted to ignore the true negative cases as well as to be highly susceptible to estimator bias (Powers, 2011). Needless to say, no single outcome metric can be equally optimal in all contexts.

Extending from the setting of healthy-diseased classification to the *multi-class setting* (e.g., comparing healthy, schizophrenic, bipolar, and autistic participants) injects ambiguity into the interpretation of accuracy scores. Rather than reporting mere better-than-chance findings in StLe analyses, it becomes more important to evaluate the F_1_, precision and recall scores for each class to be predicted in the brain scans (e.g., Brodersen et al., 2011b; Schwartz et al., 2013). It is important to appreciate that the sensitivity/specificity metrics, perhaps more frequently reported in ClSt communities, and the precision/recall metrics, probably more frequently reported in StLe communities, tell slightly different stories about identical neuroscientific findings. In fact, sensitivity equates with recall. Specificity does however not equate with precision. Further, a ClSt view on the StLe metrics would be that maximum precision corresponds to absent Type I errors (i.e., no false positives), whereas maximum recall corresponds to absent false negatives (i.e., no Type II errors). Again, Type I and II errors are related to the entirety of data points in a ClSt regime and prediction is only evaluated on a test data split of the sample in an StLe regime. Moreover, many empirical sciences usually aggregate results in *ROC* (receiver operating characteristic) curves plotting sensitivity against specificity scores, whereas other scientific domains tend to report analogous yet different *recall-precision curves* instead (Altman and Bland, 1994; Davis and Goadrich, 2006; Demšar, 2006).

Finally, StLe-minded investigators use *learning curves* (Abu-Mostafa et al., 2012; Murphy, 2012) as an important diagnostic tool for empirical estimates of the *sample complexity*, that is, the achieved model fit and prediction accuracy as a function of the available sample size n. For increasingly bigger subsets of the training set, a classification algorithm is trained on that current share of the training set and then evaluated for accuracy on the always-same test set. Across subset instances, simple models display relatively high in-sample error because they can not approximate the target function very well (underfitting) but exhibit good generalization to unseen data with relatively low out-of-sample error. Yet, complex models display relatively low in-sample error because they adapt too well to the data (overfitting) with difficulty to extrapolate to newly sampled data with high out-of-sample error. Put differently, a big gap between high in-sample and low out-of-sample performance is typically observed for high-variance models, such as artificial neural network algorithms or random forests. These performance metrics from different data splits often converge for high-bias models, such as linear support vector machines and logistic regression.

In sum, the ClSt and StLe communities rely on diagnostic metrics that are largely incongruent and may therefore not lend themselves for direct comparison in all practical analysis settings.

## Case study four: out-of-sample generalization and subsequent classical inference

Vignette: The investigator is interested in potential differences in brain volume that are associated with an individual's age (*continuous target variable*). A LASSO (*often considered as StLe arsenal*) is computed on the voxel-based morphometry data from the brain's gray matter of the 1,200-subject HCP release (Human Connectome Project; Van Essen et al., 2012). This L1-penalized residual-sum-of-squares regression performs automatic variable selection (i.e., *effectively eliminates coefficients by setting them to zero*) on all gray-matter voxels' volume information in a *high-dimensional regime* (i.e., no mass-univariate analysis). Assessing *generalization* performance of different sparse models using 5-fold cross-validation yields the non-zero coefficients for few brain voxels whose volumetric information is most *predictive* of an individual's age.

Question: How can the investigator perform *classical inference* to know which of the gray-matter voxels selected to be predictive for biological age are *statistically significant*?

This is an important concern because most statistical methods currently applied to large datasets perform some explicit or implicit form of variable selection (Jenatton et al., 2011; Committee on the Analysis of Massive Data et al., 2013; Hastie et al., 2015). There are even many different forms of preliminary selection of variables before performing significance tests on them. First, LASSO is a widely used estimator in engineering, compressive sensing, various “omics” branches and other sciences, where it is often applied without an additional significance test. Beyond neuroscience, generalization-approved statistical learning models are routinely solving a diverse set of real-world challenges. This includes algorithmic trading in financial markets, fraud detection in credit card transactions, real-time speech translation, SPAM filtering for e-mails, face recognition in digital cameras, and piloting self-driving cars (Jordan and Mitchell, 2015; LeCun et al., 2015). In all these examples, statistical learning algorithms successfully generalize to unseen, later acquired data and thus tackle the problem heuristically without classical significance test on specific variables or for overall model performance.

Second, the LASSO has been introduced as an elegant solution to the combinatorial problem of what subset of gray-matter voxels is sufficient for predicting an individual's age by *automatic variable selection* (Tibshirani, 1996). Computing voxel-wise *p*-values would recast this high-dimensional pattern-learning setting (i.e., considering all brain voxels at once) into a mass-univariate hypothesis-testing problem (i.e., considering one voxel after the other) where relevance would be computed independently for each voxel and correction for multiple comparisons would become necessary. Yet, recasting into the mass-univariate setting would ignore the sophisticated selection process that led to the predictive model with a reduced number of variables (Wu et al., 2009). Put differently, variable selection via the LASSO is itself a stochastic process that is however not accounted for by the theoretical guarantees of classical inference for statistical significance (Berk et al., 2013). Put in yet another way, data-driven model selection is corrupting the null hypothesis of classical statistical inference because the sampling distribution of the parameter estimates is altered. The important consequence is that naïve classical inference expects a non-adaptive model chosen before data acquisition and can therefore not be readily used along LASSO in particular or arbitrary selection procedures in general^6^.

Third, the portrayed conflict between more exploratory model selection by cross-validation (StLe) and more confirmatory classical inference (ClSt) is currently at the frontier of statistical development (Loftus, 2015; Taylor and Tibshirani, 2015). New methods for so-called *post-selection inference* (or *selective inference*) allow computing *p*-values for a set of features that have previously been chosen to be meaningful predictors by some criterion, one example being sparsity-incuding prediction algorithms such as LASSO. According to the theory of ClSt, the statistical model is to be chosen before visiting the data. Classical statistical tests and confidence intervals therefore become invalidated and the *p*-values become optimistically biased (Berk et al., 2013). Consequently, the association between a predictor and the target variable must be even stronger to certify the same level of significance. Selective inference for modern adaptive regression thus replaces loose *naïve p-values* by more rigorous *selection-adjusted p-values*. As an ordinary null hypothesis can hardly be adopted in this adaptive testing setting, conceptual extension is also prompted on the level of ClSt theory itself (Hastie et al., 2015). For instance, closed-form solutions to adjusted classical inference after variable selection already exist for principal component analysis (Choi et al., 2014) and forward stepwise regression (Taylor et al., 2014). Moreover, a simple alternative to formally account for preceding model selection is *data splitting* (Cox, 1975; Wasserman and Roeder, 2009; Fithian et al., 2014), which is frequent practice in genetics (e.g., Sladek et al., 2007). In this procedure, the variable selection procedure is computed on one data split and *p*-values are computed on the remaining second data split. However, such data splitting is not always possible and will incur power losses.

In sum, in many analysis settings, the same data should typically not be used to first apply supervised learning algorithms for automatic selection of the most predictive variables and to then test for statistical significance of the variables already found to be most predictive based on these data points. The recent developments for post-selection inference can be viewed as an attempt to reconcile certain aspects of how the StLe and ClSt paradigms draw conclusions from data.

## Case study five: classical inference and subsequent out-of-sample generalization

Vignette: The investigator is interested in potential brain structure differences that are associated with an individual's gender (*categorical target variable*) in the voxel-based morphometry data of the 1,200-subject HCP release (Human Connectome Project; Van Essen et al., 2012). First, the >100,000 voxels per brain scan are reduced to the most important 10,000 voxels to lower the computational cost and facilitate estimation of a prediction model. To this end, ANOVA (*univariate test for statistical significance belonging to ClSt*) is initially used to obtain a ranking of the most relevant 10,000 features from the gray matter. This selects the 10,000 out of the original >100,000 voxel variables with highest variance explaining volume differences between males and females (i.e., *the gender information associated with each brain scan is used in the univariate test*). Second, support vector machine classification (“*multivariate” pattern-learning algorithm belonging to StLe*) is performed by cross-validation on a feature space with the 10,000 preselected gray-matter measurements to predict the gender from each subject's brain scan.

Question: Is an analysis pipeline with *univariate classical inference* and subsequent *high-dimensional prediction* valid if both steps rely on gender as the target variables?

The implications of feature engineering procedures applied before training a learning algorithm is a frequent concern and can require subtle answers (Guyon and Elisseeff, 2003; Kriegeskorte et al., 2009; Lemm et al., 2011; Hanke et al., 2015). In most applications of predictive models the large majority of brain voxels will not be very informative (Brodersen et al., 2011a). The described scenario of *dimensionality reduction* by feature selection to focus prediction is clearly allowed under the condition that the ANOVA is not computed on the entire data sample. Rather, the initial identification of voxels explaining most variance between the male and female individuals should be computed only on the training set in each cross-validation fold. In the training set and test set of each fold the same identified candidate voxels are then regrouped into a feature space that is fed into the support vector machine algorithm. This ensures an identical feature space for model training and model testing but its construction only depends on structural brain scans from the training set. Generally, voxel preprocessing performed before model training is authorized if the feature space construction is not influenced by properties of the concealed test set. In the present scenario, the Vapnik-Chervonenkis bounds of the cross-validation estimator are therefore not loosened or invalidated if class labels have been exploited for feature selection or depending on whether the feature selection procedure is univariate or multivariate (Abu-Mostafa et al., 2012; Shalev-Shwartz and Ben-David, 2014). Put differently, the cross-validation procedure simply evaluates the entire prediction process including the automatized and potentially nested dimensionality reduction approaches. In sum, in an StLe regime, using class information during feature preprocessing for a cross-validated supervised estimator is not an instance of *data-snooping* (or *peeking*) if done exclusively on the training set (Abu-Mostafa et al., 2012).

At the core of this explanation is the goal of cross-validation to yield *out-of-sample estimates*. In stark contrast, remember that null-hypothesis testing yields *in-sample estimates* as it needs all available data points to take its decision. Using the class labels for a variable selection step just before null-hypothesis testing on a same data sample would invalidate the null hypothesis (Kriegeskorte et al., 2009, 2010). Consequently, in a ClSt regime, using class information to select variables before null-hypothesis testing will incur an instance of *double-dipping* (or *circular analysis*). This also occurs when, for instance, first correlating a behavioral measure with brain activity and then using the identified subset of brain voxels for a second correlation analysis with that same behavioral measurement (Lieberman et al., 2009; Vul et al., 2009). In this scenario, voxels are submitted to two statistical tests with the same goal in a nested, non-independent fashion (Freedman, 1983). This corrupts the *validity of the null hypothesis* on which the reported test results conditionally depend.

Regarding interpretation of the results, the classifier will miss some brain voxels that only carry relevant information when considered in voxel ensembles. This is because the ANOVA filter has kept voxels that are independently relevant (Brodersen et al., 2011a). Univariate feature selection in high-dimensional brain scans may therefore systematically encourage model selection (i.e., each weight combination equates with a model hypothesis from the classifier's function space) that is not tuned to neurobiological meaningfulness. Concretely, in the discussed scenario the classifier learns *complex patterns between voxels that were previously chosen to be individually important*. This may considerably weaken the interpretability and conclusions on “whole-brain multivariate patterns”. Remember also that variables that have a *statistically significant association* with a target variable do not necessarily have good *generalization performance*, and vice versa (Shmueli, 2010; Lo et al., 2015; Bzdok and Yeo, 2017). On the upside, it is frequently observed that the combination of whole-brain univariate feature selection and linear classification is among the best approaches if the primary goal is maximizing *prediction performance* as opposed to maximizing *interpretability*.

Finally, it is interesting to consider that ANOVA-mediated feature selection to a subset of *p* < 500 voxel variables would reduce the “wide” neuroimaging data (“*n* < < *p*” setting) down to “long” neuroimaging data with fewer features than observations (“*n* > *p*” setting) given the *n* = 500 subjects (Wainwright, 2014). This allows recasting the StLe regime into a ClSt regime in order to fit a GLM and perform classical statistical tests instead of training a predictive classification algorithm (Brodersen et al., 2011a).

In sum, in many analysis settings, prediction algorithms can be trained after choosing the input variables most significantly associated with an explanatory target variable if the initial classical inference (*p*-values) is performed only in the training set and the ensuing evaluation of algorithm generalization (prediction performance) is performed on the independent test set.

## Case study six: structure discovery by clustering algorithms

Vignette: Each functionally specialized region in the human brain probably has a unique set of long-range connections (Passingham et al., 2002). This notion has prompted connectivity-based parcellation methods in neuroimaging that segregate an ROI (can be locally circumscribed or brain global; Eickhoff et al., 2015) into distinct cortical modules (Behrens et al., 2003). The whole-brain connectivity for each ROI voxel is computed and the voxel-wise connectional fingerprints are submitted to a clustering algorithm (i.e., *individual brain voxels in the ROI are the elements to group; the connectivity strength values are the features of each element for similarity assessment*). The investigator wants to apply connectivity-based parcellation to the fusiform gyrus to segregate this ROI into cortical modules that exhibit similar connectivity patterns with the rest of the brain and are, thus potentially, functionally distinct. That is, voxels within the same cluster in the ROI will have more similar whole-brain connectivity properties than voxels from different clusters in the fusiform gyrus.

Question: Is it possible to decide whether the obtained brain *clusters* are *statistically significant*?

In essence, the aim of connectivity-guided brain parcellation is to find useful, simplified structure by imposing circumscribed compartments on brain topography (Yeo et al., 2011; Smith et al., 2013; Frackowiak and Markram, 2015). This is typically achieved by using k-means, hierarchical, Ward, or spectral clustering algorithms (Thirion et al., 2014; Eickhoff et al., 2015). Putting on the ClSt hat, an ROI clustering result would be deemed statistically significant if the obtained data are incompatible with the null hypothesis that the investigator seeks to reject (Everitt, 1979; Halkidi et al., 2001). Choosing a test statistic for clustering solutions to obtain *p*-values is difficult (Vogelstein et al., 2014) because of the need to find a meaningful null hypothesis to test against (Jain et al., 1999). Put differently, for classical inference based on statistical hypothesis testing one may need to pick an arbitrary null hypothesis to falsify. It follows that neither the ClSt notions of effect size and power do seem to apply in the case of brain parcellation (also a frequent question by paper reviewers). Instead of classical inference to formally *test* for a particular structure in the clustering results, the investigator actually needs to resort to exploratory approaches that discover and assess structure in the neuroimaging data (Tukey, 1962; Efron and Tibshirani, 1991; Hastie et al., 2001). Although statistical methods span a continuum between the two poles of ClSt and StLe, finding a clustering model with the highest fit in the sense of explaining the regional connectivity differences at hand is perhaps more naturally situated in the StLe community.

Putting on the StLe hat, the investigator realizes that the problem of brain parcellation constitutes an *unsupervised* learning setting without any target variable y to predict (e.g., cognitive tasks, the age or gender of the participants). The learning problem does therefore not consist in estimating a supervised predictive model y = f(X), but to estimate an unsupervised descriptive model for the connectivity data X themselves. Solving such unsupervised estimation problems is generally recognized to be ill-posed because it is generally unclear what the best way is to quantify how well relevant structure has been captured and what notion of “relevance” is most pertinent (Hastie et al., 2001; Ghahramani, 2004; Bishop, 2006; Shalev-Shwartz and Ben-David, 2014). In clustering analysis, there are many possible transformations, projections, and compressions of X but there is usually no unique criterion of optimality that clearly suggests itself. On the one hand, the “true” *shape of clusters* is unknown for most real-world clustering problems, including brain parcellation studies. On the other hand, finding an “optimal” *number of clusters* represents an unresolved issue (*cluster validity problem*) in statistics in general and in brain neuroimaging in particular (Jain et al., 1999; Handl et al., 2005). In other words, “the clustering problem is inherently ill posed, in the sense that there is no single criterion that measures how well a clustering of data corresponds to the real world” (Goodfellow et al., 2016). Evaluating the adequacy of clustering results is therefore conventionally addressed by applying different *cluster validity criteria* (Thirion et al., 2014; Eickhoff et al., 2015). These heuristic metrics are useful and necessary because clustering algorithms will always find some subregions in the investigator's ROI, that is, find relevant structure with respect to the particular optimization objective of the clustering algorithm whether such structure truly exists in nature or not. The various clustering validity criteria, possibly based on information theory, topology, or consistency (Eickhoff et al., 2015), typically encourage cluster solutions with low within-cluster and high between-cluster differences according to a certain notion of optimality. Given that the notions of optimality are not coherent with each other (Shalev-Shwartz and Ben-David, 2014; Thirion et al., 2014), investigators should evaluate cluster findings and choose the cluster number by relying on a set of complementary cluster validity criteria, such as reproducibility and goodness of fit or bias and variance.

Evidently, the discovered set of connectivity-derived clusters only represent hints to candidate brain modules. Their “existence” in neurobiology requires further scrutiny (Thirion et al., 2014; Eickhoff et al., 2015). Nevertheless, such clustering solutions provide important means to narrow down high-dimensional neuroimaging data. Preliminary clustering results broaden the space of research hypotheses that the investigator can articulate. For instance, unexpected discovery of a candidate brain region (cf. Mars et al., 2012; zu Eulenburg et al., 2012) can provide an argument for future experimental investigations. Brain parcellation can thus be viewed as an exploratory unsupervised method outlining relevant structure in neuroimaging data that can subsequently be tested as research hypotheses in targeted future neuroimaging studies on classical inference or out-of-sample generalization.

In sum, in most analysis settings, quantifying the importance of clustering solutions is inherently ill-posed because, without an explanatory target variable, many different low-dimensional reexpressions of high-dimensional input data can be useful. Choosing the right variant among the possible dimensionality reductions by clustering algorithms alone can typically not be done based on extrapolation metrics from ClSt (*p*-values, effect size, power) or StLe (out-of-sample prediction performance, learning curves).

## Conclusion

A novel scientific fact about the brain is only valid in the context of the complexity restrictions that have been imposed on the studied phenomenon during the investigation (Box, 1976). Tools of the imaging neuroscientist's statistical arsenal can be placed on a continuum between *classical inference* by hypothesis falsification and increasingly used *out-of-sample generalization* by extrapolating complex patterns to independent data (Efron and Hastie, 2016). While null-hypothesis testing has been dominating academic milieus in the empirical sciences and statistics departments for several decades, statistical learning methods are perhaps still more prevalent in data-intensive industries (Breiman, 2001; Vanderplas, 2013; Henke et al., 2016). This sociological segregation may contribute to the existing confusion about the mutual relationship between the ClSt and StLe camps in application domains such as imaging neuroscience. Despite the incongruent historical trajectories and theoretical foundations, both statistical cultures aim at inferential conclusions by extracting new knowledge from data using mathematical models (Friston et al., 2008; Committee on the Analysis of Massive Data et al., 2013). However, an observed effect in the brain with a statistically significant *p*-value does not in all cases generalize to future brain recordings (Shmueli, 2010; Arbabshirani et al., 2017; Yarkoni and Westfall, 2017). Conversely, a neurobiological effect that can be successfully captured by a learning algorithm as evidenced by out-of-sample generalization does not invariably entail a significant *p*-value when submitted to null-hypothesis testing. The distributional properties of brain data important for high statistical significance and for high prediction accuracy are not identical (Efron, 2012; Lo et al., 2015; Arbabshirani et al., 2017). The goal and permissible conclusions of a neuroscientific investigation are therefore conditioned by the adopted statistical framework (cf. Feyerabend, 1975). Awareness of the *prediction-inference distinction* will be criticial to keep pace with the increasing information detail of neuroimaging data repositories (Eickhoff et al., 2016; Bzdok and Yeo, 2017). Ultimately, statistical inference is not a uniquely defined concept.

## Author contributions

The author confirms being the sole contributor of this work and approved it for publication.

### Conflict of interest statement

The author declares that the research was conducted in the absence of any commercial or financial relationships that could be construed as a potential conflict of interest.

## Footnotes

^1^“Data Science and Statistics: different worlds?” (Panel at Royal Statistical Society UK, March 2015) (https://www.youtube.com/watch?v=C1zMUjHOLr4”)

^2^“50 years of Data Science” (David Donoho, Tukey Centennial workshop, USA, September 2015)

^3^“Are ML and Statistics Complementary?” (Max Welling, 6th IMS-ISBA meeting, December 2015)

^4^In the supervised setting, there is no a priori distinction between learning algorithms evaluated by out-of-sample prediction error. In the optimization setting of finite spaces, all algorithms searching an extremum perform identically when averaged across possible cost functions. (http://www.no-free-lunch.org/)

^5^“If you torture the data enough, nature will always confess.” (Coase R. H., 1982 How should economists choose?)

^6^“Once applied only to the selected few, the interpretation of the usual measures of uncertainty do not remain intact directly, unless properly adjusted.” (Yoav Benjamini)
